# A Study of Repair Mortars for Restoration of Wall Painted Plasters in a Hypogeum Rock-Cut Church of Matera (Southern Italy)

**DOI:** 10.3390/ma16165715

**Published:** 2023-08-21

**Authors:** Manuel Giandomenico, Filippo Edoardo Capasso, Sokol Muca, Maria Carolina Gaetani, Giovanni Quarta, Sara Iafrate, Davide Melica, Angela Calia

**Affiliations:** 1 Independent Researcher, Gioia del Colle, 70023 Bari, Italy; giandomanuel95@outlook.it; 2 Independent Researcher, 00183 Rome, Italy; filippoedoardo.capasso@gmail.com; 3 Independent Researcher, 13100 Vercelli, Italy; sokolmuca00@gmail.com; 4 Italian Ministry of Culture, Istituto Centrale per il Restauro, 00153 Rome, Italy; cgaetani@libero.it (M.C.G.); sara.iafrate@cultura.gov.it (S.I.); 5 ISPC-CNR, Institute of Heritage Sciences, Italian National Council of Research, 73100 Lecce, Italy; giovanni.quarta@cnr.it; 6 Consulenza e Diagnostica per il Restauro e la Conservazione Enterprise, Copertino, 73043 Lecce, Italy; info@diagnosticarestauro.it

**Keywords:** air lime mortars, calcareous aggregate, silica aggregate, microstructure, physical–mechanical performance, salt ageing

## Abstract

Several lime mortars for the repair of painted plasters of the rock-cut church of Ss. Pietro and Paolo in Matera were studied. They were designed taking into account both aesthetic criteria that need to be fulfilled in the field of paintings restoration, and physical–mechanical compatibility with the original materials on site, i.e., the pre-existing plasters and the supporting rock. Mixes with calcareous and silica aggregates, based on different grain size proportions, were prepared to fill missing portions of the original painted plaster. The effects of the mineralogical nature and size of the aggregates on the characteristics and properties of the mixes were investigated in relation to the microstructure, physical–mechanical features and resistance to salt ageing. At the end of the experimental campaign, the overall performance was evaluated.

## 1. Introduction

Conservation of painted plasters in cultural heritage deserves great attention as regards the choice of new materials to be employed. Especially for wall paintings in rupestrian settlements, specific and unique challenges for their conservation derive from the complex physical, chemical and biological interactions between the rock-cut context, the surrounding environment and the artefacts within it [1]. Well-confined underground environments generally present microclimatic stability due to their relative isolation from the outside, which inhibits the degradation processes promoted by variable thermo-hygrometric conditions [2,3,4]. Significant decay may be triggered when subterranean sites are connected to the outside, and thermo-hygrometric values are affected by seasonal periods [5]. In particular, salt damage may occur due to salts transported in water solutions that may penetrate the plaster’s pore network [6,7]. Original plasters often show whitening caused by salt crystallization on the surface. In the most severe cases, they are affected by decohesion and scaling, as well as lifting and flaking of pictorial layers caused by the crystallization pressures of salts growing within the material under the surface [8], and these phenomena are often observed right next to mortars applied in previous restoration works. Loss of plaster in more or less extended areas makes grouting operations necessary to fill the lacunae. In general, the choice of repair mortars should be made taking into account the compatibility with both the substrate and the pre-existing plasters under the coloured layers [9]. The compatibility between the restoration materials and the original ones, investigated under chemical–petrographic, mechanical and physical aspects, is one of the fundamental requirements for the formulation of suitable repair mortars [10,11,12]. Chemical compatibility means that restoration mortars should have a composition similar to that of the original plaster and not contain products that may trigger degradation processes in the pre-existing materials, such as soluble salts. Physical compatibility should ensure that new mortars do not act as a barrier to the movement of water, humidity and saline solutions, but do constitute a preferential path for their transfer to the surface. Mechanical compatibility affects mortar reversibility and is characterized by lower mechanical properties than those of the original plaster, so that any stresses would impact the grouting as sacrificial material rather than the original plaster. Along with these necessary constraints, durability should also be considered even if the sacrificial function of the new materials is maintained. Finally, aesthetical factors also need to be respected according to the restoration principles and theory.

Depending on these requirements, it is clear that when mortars for restoration are designed, particular attention should be paid to a variety of factors that influence the resulting performance. In primis, porosity—which is highly dependent on the components chosen for the production of a mortar, including the binder, the aggregate and the amount of water in the mix—is the characteristic that influences physical and mechanical compatibility the most [13]. Furthermore, pore sizes determine the effects of salt crystallization and, thus, mortar’s durability [14]. The aggregates also play a fundamental role in determining the mechanical properties of the mortar, depending on its nature, quantity and size [15,16,17,18].

Historic plasters are generally made of air lime binder. Therefore, materials that are at least similar in composition and properties to the original ones should be used for restoration work, according to the recommendations of international bodies, such as ICOMOS or ICCROM [19,20]. However, for several reasons, reproducing historical mortars is not an easy task. First, it is difficult to exactly determine the compositional ratio, which is one of the factors that decisively influence the characteristics and the performance of air lime mortars [21]. A further limit in re-proposing mortars similar to the historic ones is that traditional know-how in the manufacture and application of lime mortars has mostly been lost, as these mortars have been phased out in building technology since the advent of hydraulic lime and cement, whose incompatibility with traditional materials has been well established [22,23].

**Figure 1:**
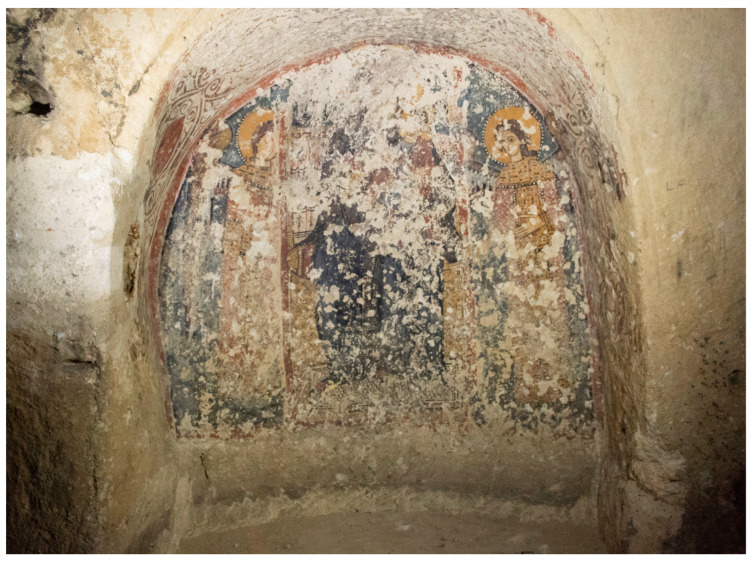
One of the wall paintings preserved in the Ss. Pietro and Paolo rock-cut crypt, representing the Madonna and Child Enthroned and Two Archangels.

In this paper, we present a study aimed at designing suitable repair mortars for the conservation of hypogeal wall paintings. The study was carried out as part of a thesis project at Istituto Centrale per il Restauro of Matera, which dealt with the conservation of the painted plasters in the hypogeal crypt of Ss. Pietro and Paolo in the Church of San Francesco (Figure 1), one of the most ancient sites within the UNESCO rupestrian settlement of Matera (Southern Italy). Rock-cut painted caves are important elements of rupestrian art [24,25]. They are widespread in Southern Italy, with an impressive example in the Matera site, where large rupestrian settlements developed, especially during the Middle Ages [26], in the form of caves excavated in the area’s soft calcarenite rock outcropping [27]. These caves were often religious and devotional sites, so their walls were decorated with painted plaster, whose unicity makes the rupestrian cultural heritage worth preserving as an example of the history and the cultural identity of the community.

Already available mortars for specific use in the restoration of wall painted plasters are very scarce. Some repair mortars are suggested for the restoration of plasters, and generally they are premixed products, based on formulations including specific types and dimensional ranges of aggregates, which can hardly guarantee the required aesthetical compatibility with the pre-existing wall painted materials. Additives also contained in these formulations, generally consisting of organic compounds, can be the feeding ground for biodeteriogens, which very often affect wall paintings in hypogeal environments; thus, they are not adequate in these contexts. Moreover, it should be noted that unlike the mortars certified in the construction sector and responding to well-defined prescriptions, at present no international guidelines or standards exist for qualifying mortars in relation to the peculiar requirements for use in the restoration field. This reflects the difficulty of making a reliable choice of commercial products on the basis of the technical information reported in the corresponding data sheets. For example, in most cases, the content of soluble salts is reported only in qualitative terms, i.e., absent, low or very low content. Where quantitative amounts are declared, there are often no exhaustive data on the salt species, nor indication of the methodologies used for the measurements. These shortcomings involve a significant lack of information to judge the safety of the new products for restoration work, especially if we consider that commercial lime mortars for restoration very often also contain hydraulic binders, which may be a source of soluble salts. Very often, the mechanical strength is also higher than that required for compatible application to specific historical artefacts.

Moving from the above-mentioned criticalities related to commercial products, air lime mortars with calcareous and silica aggregates and with different granulometric assortments were prepared. Attention was paid to lime putty as a binder, to take into account the need for preservation of historical–artistic building heritage using compatible traditional lime materials. Moreover, the new mortars were set on the basis of the knowledge of specific aspects of the original plaster and the underlying rock, as well as aesthetic restoration requirements. Microstructure features, physical–mechanical properties and resistance to salt ageing were investigated using several analyses and tests for the characterization of the different mixes and evaluated for the screening of the most suitable mortars.

## 2. Materials and Methods

### 2.1. The Pre-Existing Rock and Plaster

**Figure 2:**
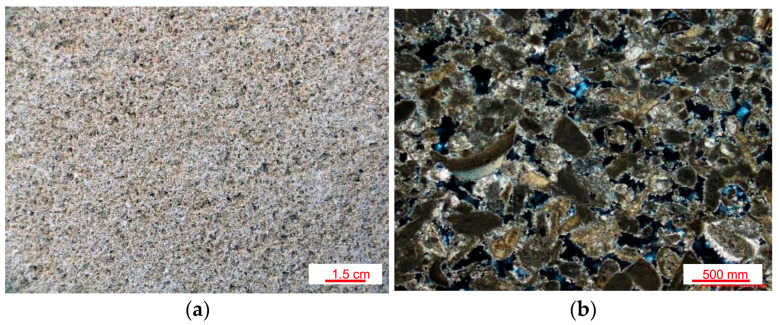
(**a**) Macroscopic appearance of the calcarenitic cut rock; (**b**) thin-section micrograph under polarized light and cross nicols of the same cut rock.

The mediaeval crypt of Ss. Pietro and Paolo in Matera is a church excavated within a Plio-Plestocenic calcarenite (Gravina Calcarenite geological formation), locally named “calcareous tuff”. The rock has a pale yellow colour and is almost exclusively made of fossil remains bound by poor calcite cement (Figure 2a,b). It is a soft rock and typically has a very high (from 40 to 50%, approximately) and coarse porosity, with prevailing pore radius sizes between 10 μm and some hundreds of micrometres, which promotes high capillary water absorption up to about 700 mg/cm^2^ [28,29].

**Figure 3:**
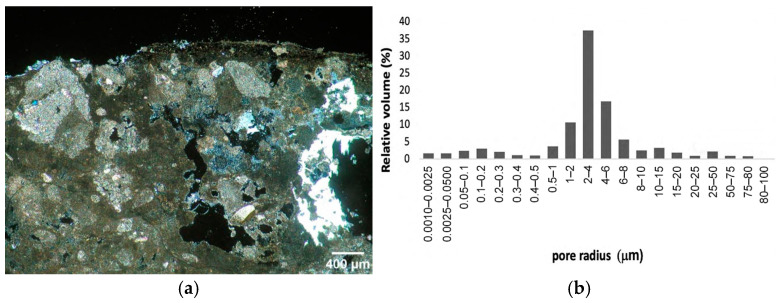
(**a**) Thin-section micrograph under polarized light and cross nicols; (**b**) porosimetric distribution of the original plaster.

The frescoes are applied on a plaster with an average thickness of 1 cm, which covers the cut rock surface. The plaster is made of air-hardening lime and calcareous aggregates coming from the grinded calcarenite of the rocky bank (Figure 3a). Clasts’ dimensions range from 100 to 900 μm, with an average size mostly between 200 and 400 µm. The binder:aggregate ratio estimated on thin sections was 1:1.5 by volume. The plaster shows dissolution phenomena at the expense of the calcite matrix and the presence of fissures and vugs often occluded by gypsum. The pictorial layer, with a thickness of about 200 µm, consists of earth pigments and whitewash. The porosity of the plaster, as determined by MIP, is 45%, with prevailing pore size radii in the range of 1–8 µm (Figure 3b) [30].

### 2.2. Experimental Materials

Two mortar types for the repair of the wall painted plasters, specific for retouchable and non-retouchable fillings of the lacunae, were designed. In the restoration field, each of these two mortars has well-defined aesthetical constraints. Retouchable mortars (R) are meant to fill missing parts of the plaster up to the painted surface level [11]. They should be white because they are destined to be painted with watercolours in order to reconstruct the missing part of the figurative pattern and have a texture similar to that of the ancient fresco, which is smooth. Non-retouchable mortars (NR) fill lacunae whose figurative reconstruction is impossible because it would imply a free interpretation of the figurative text by the restorer. In this case, grouting must be realized at a slightly lower level than the painted surface and the repair mortar is not supposed to be painted or retouched, so it should have a proper colour itself, not strongly in contrast with the painting appearance, and a proper texture, irregular and coarse [11]. As reported below, different aggregates (calcareous and silicate) were used for the two mortar types to respond to these different needs.

To address, as much as possible, the requirements of physical–mechanical compatibility with the original plasters, as well as with the underlying rock, the design of the formulations was made on the basis of some features of the pre-existing plaster and its state of conservation. In particular, considering the high porosity of the original plaster and the calcareous rock support, the design of the mortars aimed at porous and low-strength formulations.

For both experimental mortars, lime putty binder was used. It gives weaker mechanical strength than hydraulic lime [13]. Compared to hydrated lime powder, it has better workability [31] and achieves higher porosity levels and better pore size assortments [32], the latter providing a lower susceptibility to salt damage.

**Figure 4:**
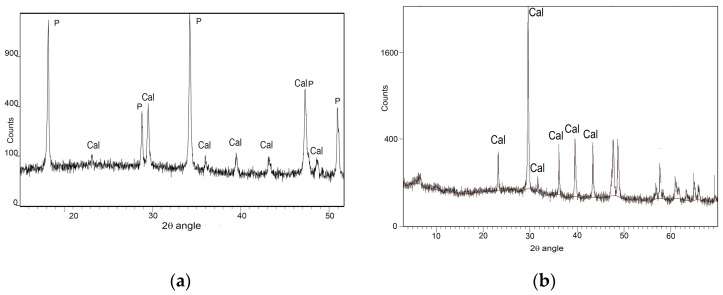

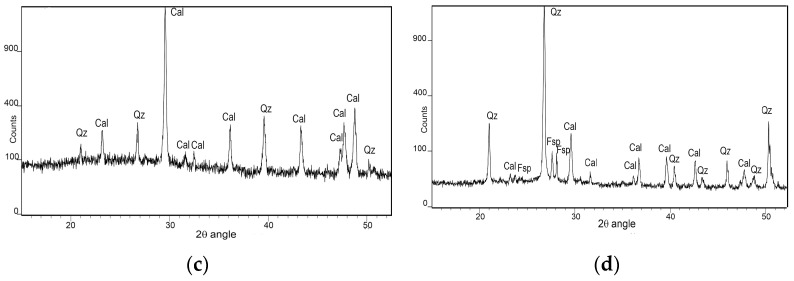
XRD spectra of (**a**) lime putty, (**b**) the whitish “tufina” aggregate (T), (**c**) the orange-coloured calcarenite aggregate (C), and (**d**) river sand (G). (Cal: calcite; P: portlandite; Qz: quartz; Fsp: feldspar).

The lime putty used (by Calceviva in Fasano, Southern Italy) is over 24 months old. It is classified as CL90-SPL [33] and is mainly composed of portlandite and calcite (Figure 4a). The following aggregates were selected: ground calcarenite (tufina, T) sourced locally from Matera, with a whitish–pale yellow colour and composed of approximately 98% CaCO_3_ (Figure 4b); ground calcarenite (carparo, C) from the nearby Puglia region, with a warm orange colour and composed of approximately 80% CaCO_3_ (Figure 4c); and river sand from Ginosa (G), near Matera (Figure 4d). The T aggregate has the same provenance as the aggregate in the original plaster within the church, namely the calcarenite from the hosting rock bank belonging to the geological outcrop of Gravina calcarenite. C is a Pleistocene variety of the soft and porous calcarenites outcropping in Southern Italy [29], while Ginosa sand comes from the Plio-Pleistocenic deposits in the area of Matera [34] and is mainly composed of quartz, chert, feldspars and a low amount of calcite. The mineralogical composition of T, C and G is shown in Figure 4b–d.

Considering that the pre-existing plaster had been weakened by the depletion of the carbonate matrix caused by dissolution phenomena promoted by water and due to salt action, as verified by microscopic observations, the ratio of binder and aggregates (B:A) was chosen to be 1:3, which was higher than 1:1.5 of the original mortar, to achieve an acceptable compromise between porosity and mechanical strength. It has been demonstrated that porosity is strongly linked to the lime content: lime-rich mortars, with a 1:1 binder/aggregate mortar, are more porous than those with more aggregates because lime is a very porous material [17]. High lime contents, such as those corresponding to 1:1 and 1:2, also determine higher mechanical strength than the lower B:A ratio of 1:3 [35], although this effect is often observed in the long run [21]. Indeed, a high binder content has a number of implications for mechanical behaviour: while it increases porosity, which reduces strength, it can also improve mechanical properties as it leads to a more continuous structure, due to fewer voids at the interface with the aggregates, and it also favours carbonation, thanks to the high air permeability [17]. On the contrary, it has been found that 1:3 mortars have mechanical strength similar to that of mortars poorer in lime, such as those with B:A ratios of 1:4 and 1:5, although the former are more porous than the latter [17,36]. Moreover, faster carbonation with less crack development in the final products has been demonstrated for long-term aged lime putty (>1 year) with B:A ratios ≤ 1:4 [35]. The ratio of binder to water was 1:3 in volume, which includes the water in the lime putty and the water added during the mixing.

#### 2.2.1. Retouchable Mortars (R)

**Figure 5:**
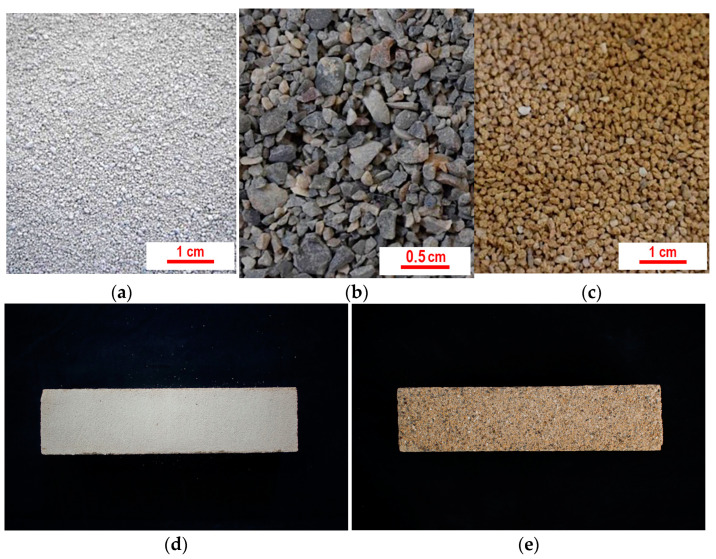
T, G and C aggregates ((**a**–**c**)**,** respectively); R and NR representative mortar samples ((**d**,**e**), respectively).

The whitish colour required for the retouchable mortars was obtained by using the pale yellow aggregate of the T type (Figure 5a). Starting from the clast sizes microscopically observed in the pre-existing plaster, ranging from 100 µm to 900 µm, the following granulometric fractions were considered: 125 µm < x < 250 µm; 250 µm < x < 500 µm; 500 µm < x < 1 mm.

**Figure 6:**
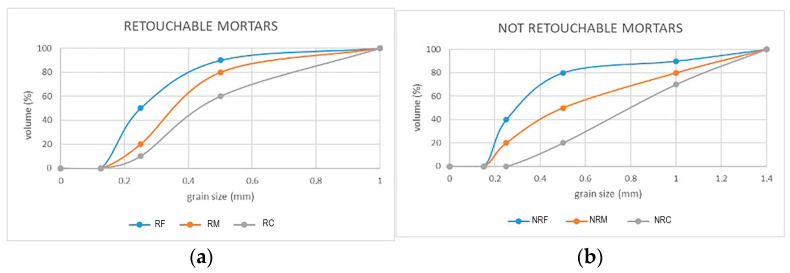
Granulometric curves for the aggregates in (**a**) retouchable mortars; (**b**) non-retouchable mortars.

Three different retouchable mortar formulations were designed by mixing different percentages of aggregate in these dimensional ranges: RF and RM corresponding to the prevailing fine (50%) and medium aggregate (60%), respectively, and RC having the highest amount of coarse aggregates (40%) and the lowest one in the finest range (Table 1, Figure 6a). The real and bulk densities of the aggregate mixes and the composition of each R mortar are reported in Table 2.

#### 2.2.2. Non-Retouchable Mortars (NR)

The T-type aggregate used in R mortars was not appropriate to fulfil the aesthetical restoration constraints for non-retouchable mortars because of the whitish colour that it produces. Therefore, yellow calcarenite (C-type) and river sand (G) were used for these mortars, as these aggregates were able to provide the proper colour and texture (Figure 5b,c). Moreover, in addition to the grain sizes adopted for the R formulations, a further dimension ranging from 1 to 1.4 mm was introduced, as medium-fine sand granulometries yielded a rough and vibrant mortar appearance. Several preliminary mix tests made it necessary to change the proportions between the granulometric ranges implemented for R mortars, and they led to the following formulations (Table 3, Figure 6b): NRF with prevailing fine to medium aggregates (80%); NRM with prevailing medium and coarse aggregates (60%); and NRC, characterized by a predominance of coarse (a) and coarse (b) aggregates (80%). The real and bulk densities of the aggregate mixes and the composition of each NR mortar are reported in Table 2.

Samples of both mortar types, with different dimensions depending on the tests, were prepared in casts. Then, they underwent 90 days of curing in laboratory conditions (20 °C and 60% RH).

### 2.3. Analyses and Tests

Several analyses and tests were conducted to investigate the mortar’s characteristics and properties.

- The mortars’ microstructure was investigated on thin sections under polarized transmitted light using optical microscopy (Eclipse LV100 Nikon);
- Porosity features were determined using mercury intrusion porosimetry (MIP). Pore size distribution (pore radii in a range from 0.001 µm to 100 µm) and the integral open porosity were measured with a Pascal 140/240 Series porosimeter from Thermo Finningan (maximum injection pressure of 200 MPa). For each mortar mix, three specimens with a volume between 2 and 3 cm^3^ were tested. The apparent density (γa) of the specimens was also measured using MIP;
- Physical behaviour under the ultrasonic pulse velocity (UPV) test was investigated according to ASTM standard D2845-05 [37]. Three cubes 4 × 4 × 4 cm for each mix were used. Before the test, they were dried in an oven at 60 °C until they reached a constant weight. The direct transmission method was employed, with the transmitter and the receiver positioned on the opposite faces of the specimens. The UPV results were expressed as the average of the measurements along the x, y, and z directions, and at each point the average of three acquisitions was considered. A Panametrics Epoch 4 Plus Ultrasonic Flaw Detector (Olympus), with a pair of ultrasonic transducers of 18 mm diameter and 1 MHz central frequency, was used for the measurements. A coupling agent (ultrasonic gel) served to improve the signal readability. The pulse velocity was calculated as the ratio of the distance between the transducers (measured using a digital calliper with a precision of 0.01 mm) and the time of flight (automatically registered by the instrument).
- The capillary water absorption test was executed on five 5 × 5 × 2 cm specimens, according to the UNI EN 15801 standard [38]. The mortars’ weights were recorded up to 5 days, that is, when the absorption reached a constant value (successive weight variations less than 0.1%). The ratios of the weight of the absorbed water to the absorbing surface area were plotted versus the square root of time (in seconds) to obtain the corresponding curves. The total water amount (Q) absorbed by the samples and the absorption coefficient (AC) were calculated.
- The water vapour permeability test was carried out on five specimens 5 × 5 × 1 cm for each mix according to the UNI EN15803 standard [39]. The test conditions were 23 °C and 50% RH. The water vapour permeability (δp) was expressed as:

δp = G × D/A × ∆p_v_ (1)

where G is the rate of the vapour flow, D is the specimen thickness, A is the surface of evaporation and Δp_v_ is the water vapour pressure difference across the specimen.

The water vapour resistance coefficient (µ) was determined as:

µ = δa/δp(2)

where δa is the water vapour permeability of air.

- Uniaxial compressive strength (UCS) and flexural strength (FS) were determined following the UNI EN 1015-11 standard [40]. The FS test was carried out on three 4 × 4 × 16 cm specimens for each mortar. The two resulting halves of each specimen, obtained from the bending rupture, were used for the UCS test. Both tests were conducted with a Controls Model 65 testing machine equipped with 15 kN load pistons.
- A microdrilling test was also performed to determine the resistance of the mortars to a rotating tip. Drilling resistance was measured using a Drilling Resistance Measurement System (DRMS) Cordless device (SINT Technology), equipped with a polycrystalline diamond-coated flat-tip drill bit of 5 mm diameter. The drilling test was performed on one 4 × 4 × 16 cm specimen for each mortar mix. Three holes per specimen were drilled up to a depth of 20 mm. The penetration force was recorded every 0.05 mm for a total of 200 acquisitions for each hole. The wearing of the drill was almost negligible for the soft lime mortars used in this study. The penetration rate and rotational speed were established after preliminary tests and were set at 20 mm/min and 50 rpm, respectively.
- Salt ageing test. There is still no commonly accepted procedure for mortar salt ageing [41,42]. The test was performed following the RILEM MSA2 recommendation [43] with some modifications, such as a lower concentration of the saline solution to reproduce more realistic conditions. Cubic specimens with sides of 4 cm were dried in an oven at 60 °C and then sealed on the lateral surfaces using Parafilm stripes in order to convey the evaporation flow only along the z-direction. Then, they were immersed 10 mm high from their base for 2 h in a 3% sodium sulphate solution, which reproduced more realistic conditions compared to higher salt concentrations [44]. After the absorption of the saline solution, they were dried on a plastic tray for 22 h at 20 °C and 50% RH in a CH250 CLIMATEST ARGOLAB climatic chamber, after which salts formed on the surface and they were removed by brush. Daily cycles were repeated over 12 weeks (84 days). After each cycle, visual observations and weight measurements were carried out, and a graph of the weight variations as a function of time was elaborated.

## 3. Results and Discussions

### 3.1. Mortars’ Microstructure and Porosity Features

**Figure 7:**
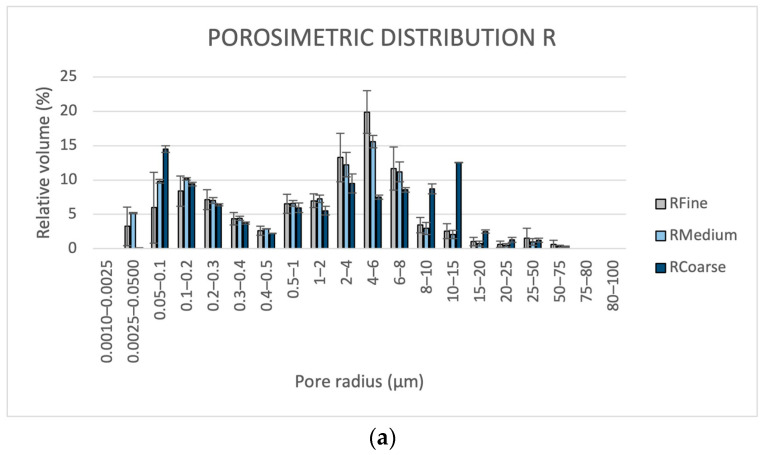

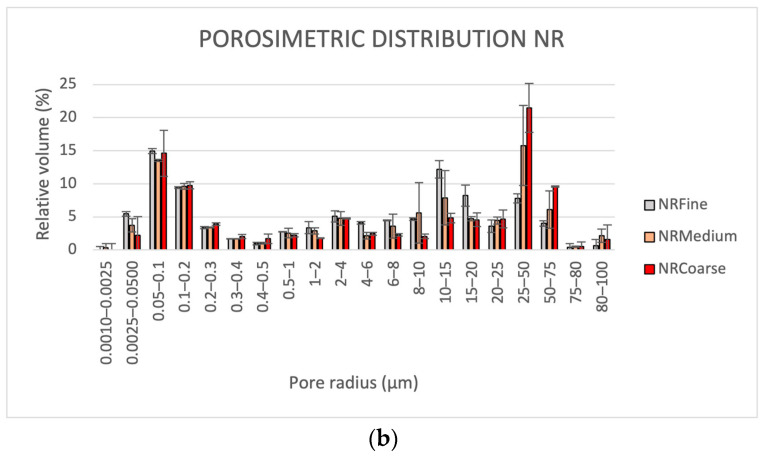
(**a**) Porosimetric distribution of R mixtures; (**b**) Porosimetric distribution of NR mixtures.

**Figure 8:**
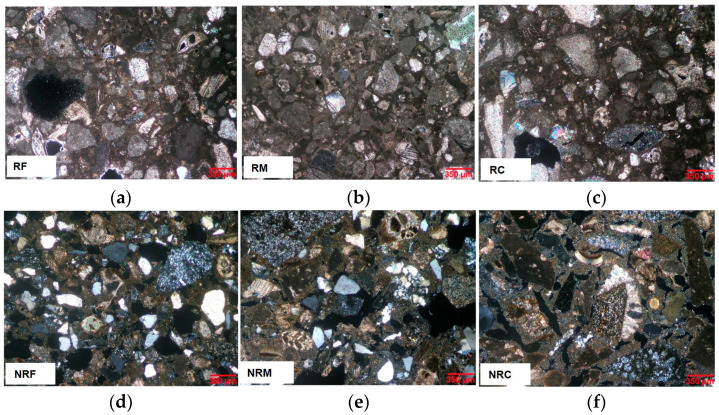
Mortars’ microstructures as observed on thin sections under polarized light, cross nicols: (**a**) RF; (**b**) RM; (**c**) RC; (**d**) NRF; (**e**) NRM; (**f**) NRC.

As illustrated in Table 4 and Figure 7 and Figure 8, different mortar microstructures and porosity features, depending on the nature and sizes of the aggregates, were detected for the formulations studied. The accessible porosities measured using MIP (Table 4) show very close values among samples of each of the two R (from 36 to 39%) and NR groups (from 33 to 35%), but higher for the former. Apparent density values were higher for NR than for R formulations.

Pore size distribution (Figure 7a) shows that the mortars of the R group have pore radii mainly between 0.0025 and 15 μm, with a bimodal trend. In particular, RF and RM have the main peak ranging from 4 to 6 μm and a second one between 0.1 and 0.2 μm. RC shows the main peak between 0.05 and 0.1 μm. Compared to RM and RF, it has a lower pore volume, in the 2–8 μm range, and a higher one from 8 to 20 μm, with a second peak centred between 10 and 15 μm.

NR formulations also have bimodal patterns (Figure 7b). They have very similar distributions under pore sizes of 4 μm, where the main size peak for each of them is between 0.05 and 0.2 μm. Over 4 μm, it can be observed that NRF has a high pore concentration in the range of 10–20 µm, while the maximum peak for NRM and NRC is set between 25 and 50 μm.

The finest pores, generally with radii lower than 1 μm, are those forming in the lime paste [45,46]. Their presence was found irrespective of the nature and grain sizes of the aggregates in both R and NR mortar formulations. As reported in Table 4, the relative volumes of pores under 1 μm in the two mortar types are similar, namely between 32% and 36% in R mortars, unless RM mix (40%), and between 33% and 36% in NR mortars.

Bigger pores starting from some microns’ size arise from the voids at the interface between the binder and the aggregates, entrapped air voids in the mix, as well as shrinkage fissures originating within the paste during drying [16,32,47].

Comparing the distributions of these pores in R and NR mortars, the former shows a higher amount in the range between 1 and <10 µm (from 37 to 58%) than NR mixes (from 14 to 20%). The latter have large pores in the range between ≥10 and 100 µm varying from 42 and 49%, while in the R mortars these pores have notably lower percentages, from 8 to 27%.

Passing from the formulations with prevailing fine grain sizes to those with prevailing coarse aggregates, an increase of the pore volume with radii ranging from 10 to 100 μm was recorded, at the expense of pores between 1 and 10 μm (Table 4). This finding was recorded for both mortar types, suggesting that the fine aggregate mixes more homogeneously with the binder, thus limiting the formation of larger fissures due to shrinkage.

According to porosimetric results, the microscopic characteristics of the mortars (Figure 7a,b) show that R samples contain notably less coarse pores due to shrinkage (from 10 to 100 μm radii) than NR. On the contrary, NRM and NRC in particular show a lot of large and ultra-large pores ranging from some tens to some hundreds of micrometres in size, respectively. These ultra-large pores fall out of the upper limit of measurement using MIP.

This occurrence has to be taken into account when evaluating the integral open porosity results from MIP analysis (Table 4). As noted before, MIP porosity values in R samples were found to be slightly higher (from 36 to 39%) than in NR samples (from 33 to 35%). Such porosity values do not include voids over 100 μm radii, which can be widely observed microscopically in NR mortars, so it is evident that porosity measured using MIP in the latter is underestimated and the corresponding values of apparent density are higher than those recorded for R mortars.

The notably different microstructures resulting for R and NR mortars, despite the same binder–aggregate ratio and mixing water, can be attributed to the nature of the aggregate. Indeed, in R mixtures, the aggregate is exclusively calcareous, while in NR mortars, only 20% is calcareous and the remaining 80% is silicate. The calcareous aggregate comes from a rock having a high open porosity (between 40% and 50%), including both intergranular and intragranular voids [29,48], part of which, especially at the intragranular level, are probably maintained in the ground fraction. The porosity of the aggregate, capable of absorbing the mixing water, could account for the lower coarse shrinkage fissures observed in R mixtures. Conversely, such a type of contribution cannot be expected from the silicate aggregates, which are mainly composed of single quartz and chert grains or tightened poly-mineral fragments with an almost absent porosity. It is a fact that in R mortars, the porosity resolved microscopically, which is of both intergranular and intragranular types, mainly ranges from some micrometres to only a few tens of micrometres, and only sometimes ultra-large shrinkage pores were observed.

In addition, widespread discontinuities were found in NR samples along the silicate grains at contact with the binder. The coarser the aggregate, the more extended these fissures along the grains, so that larger pores increase, passing from formulations with prevailing fine grain sizes to coarser ones. The lower volume of large pores observed in R mortars compared to NR ones can be attributed to the higher cohesion between the matrix and the calcareous aggregates due to their chemical affinity: this leads to a reduction in the content of the discontinuities between the lime and the aggregate [49]. According to the literature, the presence of calcareous aggregates in aerial lime mortars results in a better-quality interfacial transition zone between the aggregate and the matrix [50]. During the carbonation of the lime, the calcite of the aggregates provides nucleating sites for the crystal growth of calcite in the paste, favouring compositional continuity and good adhesion between the aggregate and the matrix [16,17]. Moreover, porous calcareous aggregate, because it has an absorbent surface, may be impregnated by the binder, thus creating a strong bond between the two mortar components, which also increases mortar resistance [15].

Finally, on the basis of the porosimetric and microscopic analyses, we can state that all the experimental mortars possess high accessible porosity values, which allows us to expect a compatible use with the very porous painted plaster and the underlying rock. The porosimetric distributions of new mortars and the original mortar (Table 4) significantly differ. On the other hand, the porous structure detected for the original plaster may also be the result of modifications due to decay over time, thus hardly being matched by the new mortars. Nonetheless, both R and NR mortars have higher pore size assortments that, in principle, may ensure balanced physical performance as regards the compatibility compared with mortars having pore concentrations in poorly extended dimensional ranges.

### 3.2. Behaviour in Water Transfer

**Figure 9:**
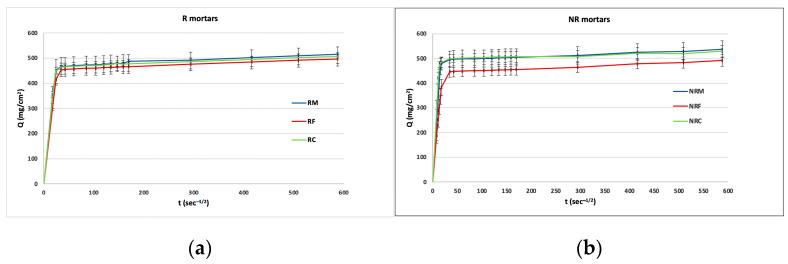
Capillary water absorption as a function of the square root of time for each formulation of (**a**) R type and (**b**) NR type.

Mean capillary absorption curves are reported in Figure 9a,b, while the coefficient of water absorption by capillarity (AC) and the total amount of water absorbed per unit area (Q) are reported in Table 5.

All the samples very quickly absorbed water by capillarity during the early steps (Figure 9a,b). In particular, the water uptake for R samples was about 90% of the total amount in the first 20′; for NR mortars, this percentage was reached after only 5′ for NRM and NRC and after 10′ for NRF.

AC values of the three R formulations were very close. NR mortars showed higher values of AC (from 22 to 34 mg/cm^2^s^−1/2^). In this case, the mortar with the prevailing fine aggregates (NRF) had a lower absorption rate.

At the end of the test, namely after 5 days, the total amounts of water absorbed were comparable for both R and NR mortars.

On the basis of the results obtained, it can be concluded that both types of mortars ensure high water transfer typical of very porous materials and comparable to the hydric properties of the excavated rock of the church site. The higher kinetics of absorption recorded in NR mortars come from the higher presence of coarse pores, which very effectively contribute to capillary uptake [51,52]. It seems that in R mortars, which are particularly rich in pores between 1 and 10 microns, the rate of absorption promoted by these pores is less effective compared to the larger ones.

Passing from prevailing coarse grain sizes to fine-graded mortar formulations, coarse porosity decreases, so the effect of the aggregate sizes of both calcareous and silicate types in reducing the kinetics of the water uptake is also evident. Finally, the contribution of the lime putty binder to the high water absorption has also to be taken into account, according to the literature, which reports higher absorption efficiency of the microstructure in mortars with lime putty compared to mortars with aerial lime [53,54].

The results of the water vapour permeability test (Table 5) show that the vapour resistance values of both R and NR formulations are not so diversified.

Unless the slightly higher values of the water vapour diffusion resistance coefficient (μ) near 10 for the formulations with prevailing medium-sized aggregates (RM and NRM), all the other mortar mixes have μ coefficient values of less than 10. According to the literature [55,56], values of μ < 10 are suitable for rendering and repair mortars, while standards [57,58] report higher acceptable values of μ ≤ 15 and μ ≤ 12, respectively.

### 3.3. UPVs and Mechanical Properties

As reported in Table 6, ultrasonic investigations show that R mortars generally have higher propagation velocities (from 1482 to 1386 m/s) compared to NR mortars (from 1180 to 947 m/s). The lowest velocities were recorded in the mixes with the prevailing coarser aggregates for both mortar types.

**Figure 10:**
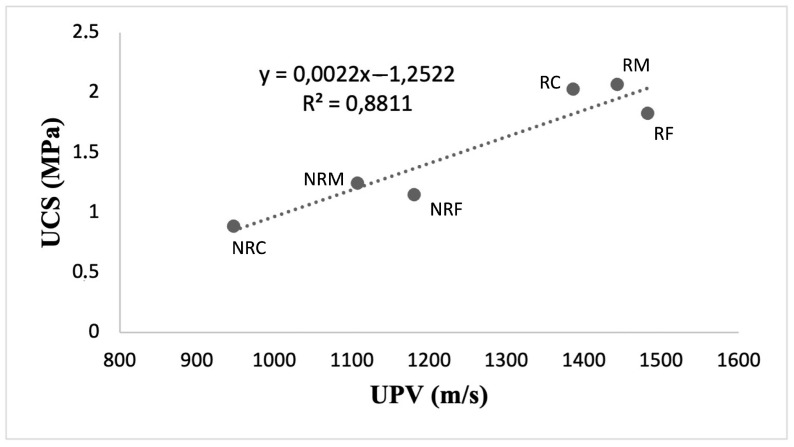
Correlation between UCS and UPV for R and NR formulations, along with the regression line (dotted line).

In a similar vein, higher compressive strengths were measured for R type. There is a good correlation (coefficient of correlation = 0.89) between the ultrasonic velocities and the compressive strength (Figure 10), as higher mechanical strength corresponds to higher ultrasonic velocities.

These results are correlated with the materials’ microstructural features. Lower UPVs and UCSs in NR mortars likely depend on higher porosity and pore sizes, as evaluated using both MIP and optical microscopy. The progressive increase in large and ultra-large pores passing from mortars with prevailing fine aggregates to mortars with coarser ones explains why the lowest velocities were found for the NRC mix.

Better bonding between the calcareous aggregate and the lime matrix, with consequent lower porosities and void sizes in R formulations, accounts for the higher propagation velocities of ultrasonic waves and higher compressive strength as well. Moreover, in these mortars, the ultrasonic velocities are influenced by the granulometric assortment of the aggregates, as mortars with fine aggregates have a more homogenous and uniform structure, with fewer largest voids than mortars made of coarse ones, and this results in higher velocity values.

The bending strength test showed significant differences between the formulations of R and NR groups, with quite lower resistance in the latter (Table 6). The coarser the grains, the more relevant these results are to the lower quality of the interfacial transition zone due to diffuse discontinuities between the silica aggregates and the carbonate binder. Indeed, the differences among FSs were negligible for R formulations, while they were more evident within the NR group.

Compressive and flexural strengths for both N and NR indicate good mechanical performance within the range reported in the literature for air-hardening lime mortars for restoration [17,59] and close to the quite high values reported by [21,32,60] and suggest a positive contribution of the lime putty to the mechanical properties of the investigated mortars [21,54].

The presence of silicate aggregates in the NR formulations also resulted in lower strengths under the microdrilling test (Table 6). The results show that R mortars, unless an RC formulation, show higher mean drilling resistance than NR mortars. The strength measured for the RC mix is less than half of the values measured for the RF and RM mixes. This behaviour could be an effect of the coarse grains, which lead to the formation of larger chips and, consequently, lower forces recorded. As for the NR mortars, no differences were observed in the values of the mean drilling resistance among the three formulations due to a prevailing effect of the discontinuities between the grains, which cause a larger propagation of the cracked zone and the formation of larger fragments during drilling [61].

### 3.4. Resistance to Salt Ageing

**Figure 11:**
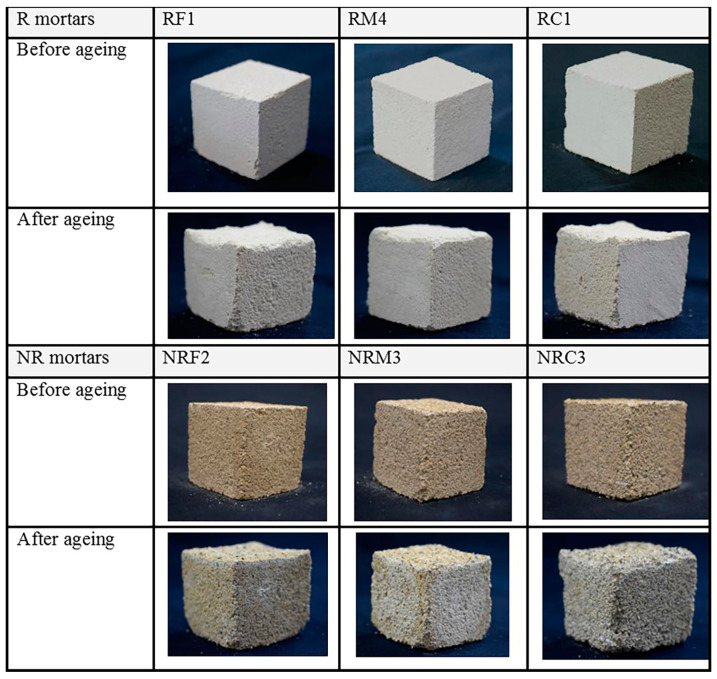
Macroscopic damage observed after salt ageing on representative samples of each R and RM formulation.

After salt ageing, all R and RM samples showed evident material losses at the evaporation surface (Figure 11).

**Figure 12:**
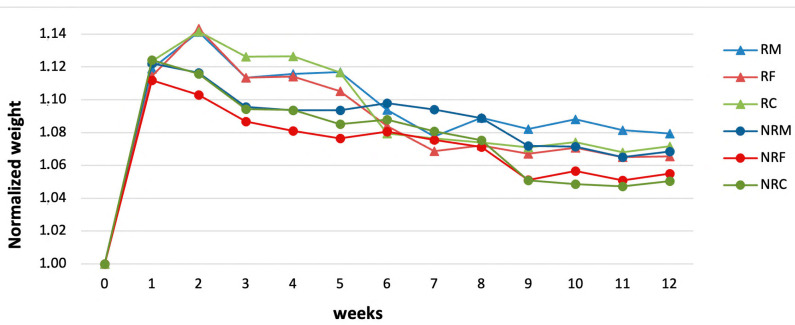
Weekly mean normalized weights for R and NR samples during salt ageing.

Weekly normalized mean weights for R samples (Figure 12) show an increase after the first two weeks, due to the salt accumulation within the porous structure. Starting from the third week, weight decreases were observed, meaning that loss of materials due to mortar decohesion, as an effect of salt damage, prevailed on salt accumulation. It is worth noting that for NR samples, weight decreases took place earlier, starting from the second week.

At the end of the test, that is, after 12 weeks, R mortars showed lower losses of material than NR mortars.

It is well known that a material’s susceptibility to salt crystallization pressures depends on pore dimensions, as the tension caused by the growth of the salt crystals is greater in small than in large pores and can overcome tensile strength, causing the breakage of pore walls [62,63]. Despite higher concentrations of large and ultra-large pores, which could lead to a lower susceptibility to salt damage, NR mortars behave worse than R, and this result can once again be attributed to the higher cohesion of the latter, which comes from the better adhesion between the grains and the lime binder. Indeed, textural characteristics play an important role in the incidence of salt damage in grain framework materials’ types [64,65]. The weight losses were recorded similarly for mortars having fine and coarse prevailing aggregates in each of the two groups, while the weight decrease was found to be slightly lower for mortar samples with prevailing medium-grained aggregates, suggesting that in these cases a better grading of the grains may have led to better compaction and void filling by lime.

## 4. Conclusions

Lime mortars with calcareous and silica aggregates (R and NR types, respectively) and with different grain size proportions for the repair of painted plasters in the rock-cut church of Ss. Pietro and Paolo in Matera were studied.

The study shows that from a physical point of view, all the formulations analysed are compatible with the characteristics of the pre-existing materials of the paintings. Both mortar types resulted in high porosity values, comparable with those of both the highly porous rock and the original plaster. MIP analyses detected pore percentages between 32 and 40%, which were likely underestimated considering the presence of ultra-large pores, detected using microscopic analysis but out of the range of porosimetric measurement. Ultra-large porosity was especially observed in mortars with silica aggregates. The behaviour of the porous system in relation to water showed that all the mixes, regardless of the grain size proportions, guarantee the transfer of water in the liquid and gaseous phase that can easily infiltrate through the rock bank in hypogeal conditions. This means that the new mortars would not create a barrier to the migration of water moving from inside the rock bank to the surface of the painted mortar. On the contrary, repair mortars will act as permeable fillers, avoiding the accumulation of water and subsequent damages at the expense of the surrounding original plaster.

Mortar microstructures were found to be different among the formulations. The presence of large pores (between 10 and 100 μm) and ultra-large pores with sizes of some hundreds of micrometres was found to be typically high in the NR mortars, which had a prevalence of silica aggregate in their composition. As microscopically observed, the ultra-large pores come from fissures along the contact surface of the aggregate grains with the calcite matrix due to a diffuse lack of adhesion between them, as well as from a higher shrinkage. On the contrary, R mortars, namely those with calcareous aggregate, had lower pore sizes, mainly between 1 and 10 μm, because of the affinity between the binder and the calcareous grains, which results in a better adhesion of these components and lower shrinkage as well, probably because porous calcareous rock fragments are more able than non-porous silica grains to absorb the mixing water.

In both types of mortars (R and NR), the effect of the aggregate size, passing from formulations with prevailing fine aggregates to formulations with prevailing coarse grains, led to an increment in pore size. The rate of water uptake increased as well, indicating the high effectiveness of coarse pores in capillary absorption, while mechanical strength decreased.

The study pointed out that the different microstructures also affected the behaviour of the mortar in relation to ultrasonic wave propagation velocities, and they did so by causing mortars with calcareous aggregate (R type) to have higher compressive and flexural strengths than those with silica aggregate (NR type). Resistance to the microdrill test also confirmed this trend. The measured mechanical properties typically denote low-strength mortars that are able to ensure a sacrificial function in repair works. The use of lime putty binder seems to provide good mechanical quality levels within the performance variability of air-hardening lime mortars, confirming that traditional materials deserve attention in the current mortar’s technology in order to obtain suitable materials to be used in the restoration field.

The two mortar types also exhibited different salt weathering resistance. In this regard, the study shows that the textural features of the mortars played a more major role than the pore dimensions. Despite the presence of large and ultra-large pores, which would lead to lower susceptibility to salt damage, under the salt ageing test, the mortars with silica aggregate had earlier and higher damage by decohesion than R mortars. The better quality of the interface between calcareous grains and lime binder makes the latter more able to contrast the pressures developed within the pores by the salts’ crystal growth. 

Finally, the overall results showed that mortar formulations of both types with prevailing medium-sized aggregates had the best performance in terms of mechanical strength and resistance to salt damage. This result was also due to the better grading of the aggregate.

The present research contributes to the knowledge on historic building restoration using traditional lime mortars, which is a promising research field with implications for sustainable conservation. This is the first step of a larger study aiming to obtain suitable mortars for restoration of wall paintings on porous and soft rocks in hypogean environments. After the application in the crypt, the selected mortars will be monitored on site over time in order to detect any changes in the optical and morphological properties of the repair mortars and the surrounding wall painted surfaces, in order to verify possible interactions over time between new plasters and the original materials of the artefact, as well as the durability of the new mortars themselves.

## Figures and Tables

**Table 1:** Aggregate size percentages for R mortars: RF (fine), RM (medium) and RC (coarse).

	RF	RM	RC
*Aggregate Size*	*Aggregate Amount (%)*		
**Coarse (a):** 500 µm–1 mm	10	20	40
**Medium:** 250 µm–500 µm	40	60	50
**Fine:** 125 µm–250 µm	50	20	10

**Table 2:** Density of the aggregate mixes and composition of each R and NR mortar.

	*Mortars’ Aggregate*		*Mortars’ Composition*		
	Real Density (kg/m^3^)	Bulk Density (kg/m^3^)	Lime Putty (kg/m^3^)	Aggregate (kg/m^3^)	* Total Water (kg/m^3^)
**RF**	2720	1.41	319	1061	276
**RM**	2720	1.33	319	997	276
**RC**	2720	1.25	319	938	276
**NRF**	2678	1.43	319	1073	276
**NRM**	2678	1.38	319	1036	276
**NRC**	2682	1.30	319	974	276

* Total water includes kneading water (10% of the aggregate volume) and free water (63% of the lime putty weight).

**Table 3:** Type, grain size and percentage amounts of aggregates in non-retouchable mortars: NRF (fine), NRM (medium) and NRC (coarse).

	NRF			NRM			NRC		
*Aggregate Size*	*Aggregate Type and Amounts (%)*								
	*C*	*G*	***(C + G)***	*C*	*G*	***(C + G)***	*C*	*G*	***(C + G)***
**Coarse (b):** 1 mm–1.4 mm	-	10	**10**	-	20	**20**	10	20	**30**
**Coarse (a):** 500 µm–1 mm	-	10	**10**	20	10	**30**	-	50	**50**
**Medium:** 250 µm–500 µm	20	20	**40**	-	30	**30**	20	-	**20**
**Fine:** 125 µm–250 µm	-	40	**40**	-	20	**20**	-	-	**-**

**Table 4:** Apparent density, open porosity values and relative volume of pores grouped in three main size ranges, along with standard deviations, for each R and NR mortar mix.

Mix	Apparent Density (g/cm^3^)	Porosity (%)	Pore Volume (%)		
			0.01–<1 µm	≥1–<10 µm	≥10–100 µm
**RF**	1.75 ± 0.02	38.8 ± 1.7	32	58	10
**RM**	1.69 ± 0.01	36.2 ± 0.1	40	53	8
**RC**	1.72 ± 0.04	38 ± 0.6	36	37	27
**NRF**	1.85 ± 0.01	33.2 ± 0.3	36	20	42
**NRM**	1.82 ± 0.06	34.7 ± 0.8	33	16	47
**NRC**	1.81 ± 0.02	33.6 ± 0.3	34	14	49
**original plaster**	1.66 ± 0.01	45.5 ± 0.5	13	74	13

**Table 5:** Mean values of absorption coefficient (AC), amounts of absorbed water by capillarity per unit area (Q) and water vapour diffusion resistance coefficient (µ) along with standard deviations for each mix of the R and NR group.

Mix	AC (mg/cm^2^s^−1/2^)	Q (mg/cm^2^)	µ
**RF**	17 ± 1	497 ± 18	8.5 ± 0.4
**RM**	19 ± 1	515 ± 6	9.6 ± 0.4
**RC**	19 ± 2	523 ± 12	8.9 ± 1.0
**NRF**	22 ± 2	492 ± 23	7.9 ± 0.7
**NRM**	31 ± 3	535 ± 34	10.1 ± 0.5
**NRC**	34 ± 7	523 ± 25	8.7 ± 0.9

**Table 6:** Ultrasonic pulse velocity (UPV), bending strength (FS), compressive strength (UCS) and drilling resistance (DR) mean values with corresponding standard deviations for R and NR mixtures.

MIX	UPV (m/s)	FS (MPa)	UCS (MPa)	DR Mean (N)
**RF**	1482 ± 79	1.82 ± 0.18	1.83 ± 0.28	4.51 ± 0.37
**RM**	1442 ± 75	2.01 ± 0.08	2.07 ± 0.16	4.77 ± 0.22
**RC**	1386 ± 52	1.81 ± 0.26	2.03 ± 0.19	2.09 ± 0.98
**NRF**	1180 ± 53	0.74 ± 0.11	1.10 ± 0.05	2.58 ± 0.51
**NRM**	1106 ± 80	0.68 ± 0.06	1.25 ± 0.19	2.55 ± 0.38
**NRC**	947 ± 68	0.48 ± 0.18	0.89 ± 0.09	2.66 ± 0.35

## Data Availability

Data are not yet available publicly.
